# Training and Service in Public Health, Nigeria Field Epidemiology and Laboratory Training, 2008 – 2014

**DOI:** 10.11694/pamj.supp.2014.18.1.4930

**Published:** 2014-07-21

**Authors:** Patrick Nguku, Akin Oyemakinde, Kabir Sabitu, Adebola Olayinka, Ikeoluwapo Ajayi, Olufunmilayo Fawole, Rebecca Babirye, Sheba Gitta, David Mukanga, Ndadilnasiya Waziri, Saheed Gidado, Oladayo Biya, Chinyere Gana, Olufemi Ajumobi, Aisha Abubakar, Nasir Sani-Gwarzo, Samuel Ngobua, Obinna Oleribe, Gabriele Poggensee, Peter Nsubuga, Joseph Nyager, Abdulsalami Nasidi

**Affiliations:** 1 African Field Epidemiology Network; 2 Federal Ministry of Health, Nigeria; 3 Ahamdu Bello University, Nigeria; 4 University of Ibadan, Nigeria; 5 Capacity Plus, Nigeria; 6 E&F Management Consult, Nigeria; 7 Global Public Health Solutions, Atlanta, USA; 8 Federal Ministry of Agriculture and Rural Development, Nigeria

**Keywords:** Public Health, field epidemiology, training, Capacity building, Nigeria

## Abstract

The health workforce is one of the key building blocks for strengthening health systems. There is an alarming shortage of curative and preventive health care workers in developing countries many of which are in Africa. Africa resultantly records appalling health indices as a consequence of endemic and emerging health issues that are exacerbated by a lack of a public health workforce. In low-income countries, efforts to build public health surveillance and response systems have stalled, due in part, to the lack of epidemiologists and well-trained laboratorians. To strengthen public health systems in Africa, especially for disease surveillance and response, a number of countries have adopted a competency-based approach of training - Field Epidemiology and Laboratory Training Program (FELTP). The Nigeria FELTP was established in October 2008 as an inservice training program in field epidemiology, veterinary epidemiology and public health laboratory epidemiology and management. The first cohort of NFELTP residents began their training on 20th October 2008 and completed their training in December 2010. The program was scaled up in 2011 and it admitted 39 residents in its third cohort. The program has admitted residents in six annual cohorts since its inception admitting a total of 207 residents as of 2014 covering all the States. In addition the program has trained 595 health care workers in short courses. Since its inception, the program has responded to 133 suspected outbreaks ranging from environmental related outbreaks, vaccine preventable diseases, water and food borne, zoonoses, (including suspected viral hemorrhagic fevers) as well as neglected tropical diseases. With its emphasis on one health approach of solving public health issues the program has recruited physicians, veterinarians and laboratorians to work jointly on human, animal and environmental health issues. Residents have worked to identify risk factors of disease at the human animal interface for influenza, brucellosis, tick-borne relapsing fever, rabies, leptospirosis and zoonotic helminthic infections. The program has been involved in polio eradication efforts through its National Stop Transmission of Polio (NSTOP). The commencement of NFELTP was a novel approach to building sustainable epidemiological capacity to strengthen public health systems especially surveillance and response systems in Nigeria. Training and capacity building efforts should be tied to specific system strengthening and not viewed as an end to them. The approach of linking training and service provision may be an innovative approach towards addressing the numerous health challenges.

## Introduction

The health workforce is one of the key building blocks for strengthening health systems[1]. There is an alarming shortage of curative and preventive health care workers in developing countries many of which are in Africa. The continent has an estimated 2.3 health care workers per 1000 population, compared with the Americas, where there are 24.8 health care workers per 1000 population[2]. Africa resultantly records appalling health indices as a consequence of endemic and emerging health issues that are exacerbated by a lack of a public health workforce. In low-income countries, efforts to build public health surveillance and response systems have stalled, due in part, to the lack of epidemiologists and well-trained laboratorians[3]. Weak surveillance systems coupled with untimely and uncoordinated response to disease outbreaks have continued to be a challenge in many African countries. Emerging pandemic threats require development of worldwide capacity for public health surveillance and response especially given the increased travel and urbanization. Good international public health surveillance and response, which is the basis of International Health Regulations (IHR) of 2005, cannot exist sustainably without good domestic surveillance and response operated by competent public health workers in core public health positions at national and sub-national levels with a focus on disease prevention. To achieve this, there is need to address several interrelated factors on human resources, disease surveillance and reporting capacity in an integrated and sustainable approach that enables the development of public health workforce capacity in order to achieve public health surveillance and response systems that have a sustainable and adaptable capacity to address evolving public health needs [4].

To strengthen public health systems in Africa, especially for disease surveillance and response, a number of countries have adopted a competency-based approach of training modeled after the >60 year old United States (U.S.) Centers for Disease Control (CDC)'s Epidemic Intelligence Service (EIS) program. EIS has been responsible for developing the U.S. public health surveillance and response systems at the Federal and State levels. In the 1980s, CDC formed a partnership with the World Health Organization (WHO) to establish the Field Epidemiology Training Programs (FETPs). These training programs (and their allied programs such as Public Health Schools Without Walls have shown to be a successful way to strengthen public health systems by providing a critical component of the public health workforce that is needed to operate public health surveillance and response systems to implement IHR (2005). Residents and staff of these programs provide services such as epidemic investigations, surveillance, surveys, and program evaluations to a country's Ministry of Health (MOH) while building competency in applied epidemiology. Increasingly, FETPs have incorporated other specialties including laboratory and management expertise. The first FETP to add a laboratory component was the Kenya Field Epidemiology and Laboratory Training Program (FELTP). Since majority of the emerging threats are of zoonotic origin necessitating a human animal collaboration, newer programs have incorporated a veterinary component to address animal health issues necessary for meaningful collaborations between the animal and human health sectors. It is estimated that the need for field epidemiologists is about 3 to 5 graduates of FETP/FELTPs per one million inhabitants in a country[4–11].

Many countries have adopted a tiered approach of training with the 2-year training of FELTP being at the apex and aimed at developing public health leaders. This is augmented with competency-based short courses to build necessary epidemiological capacity among the frontline surveillance and response staff at lower levels of the health system[12]. Nigeria with a population of over 170 million people in 36 states and 774 Local Government Areas (LGAs) suffers from several recurrent disease outbreaks including cholera, avian influenza, childhood lead poisoning and zoonotic diseases such as lassa fever[13–20]. The existing surveillance systems (animal and human) are weak[10, 21]. The Federal Ministry of Health (FMOH) is still rolling out the WHO African Regional Office Integrated Disease Surveillance and Response (IDSR) strategy, a platform tailor-made to strengthen surveillance in sub-Saharan Africa [22]. IDSR progress has been hindered by inadequate qualified personnel to operate the system [23]. The animal health sector surveillance system is operated under the National Animal Disease Information System (NADIS). NADIS has an unmet need for additional epidemiologists and laboratorians to ensure rapid detection and control of zoonotic diseases and other animal human interface issues such as food safety, environmental health and collaborative one health activities. The ever present threat of zoonoses in Nigeria is real given the experiences of H5N1 avian influenza outbreak in 2006[13]. The outbreak presented an opportunity to strengthen collaboration between the animal and human health sectors to address the diseases at human animal interface. There is need to build epidemiological capacity for both human and animal health sectors given the few trained epidemiologists in the systems to recommended international levels[4].

## Nigeria FELTP description

In January 2007, CDC initiated negotiations with the Federal Ministry of Health (FMOH) for the development of a Nigeria FELTP (NFELTP). Three outbreak investigation short courses lasting two weeks were conducted between June 2007 and October 2008. The 2-year masters program was established in October 2008 as an in-service training program in field epidemiology, veterinary epidemiology and public health laboratory epidemiology and management. This program, created as a long-term initiative within the FMOH and Federal Ministry of Agriculture and Rural Development (FMARD), aimed at training medical epidemiologists, veterinary epidemiologists and public health laboratory scientists for leadership positions in various levels of both ministries. While in training, the trainees (who are called residents) provide service as may be required to the FMOH, FMARD and respective State Ministries of Health (SMOH) and State Ministries of Agriculture and Rural Development through short and long-term field placements addressing mainly surveillance and response systems. Like FETPs and FELTPs, NFELTP is composed of a 25% didactic component and a 75% field based component. The course lasts 24 months and is offered in collaboration with two leading Nigerian universities-University of Ibadan (UI) in the southwest region and Ahmadu Bello University (ABU) in the northwest region. The first cohort of NFELTP residents began their training on 20th October 2008 and completed their training in December 2010. The second cohort was admitted on 26th October 2009. The program was scaled up in 2011 and it admitted 39 residents in its third cohort. The program has admitted residents in six annual cohorts since its inception admitting a total of 207 residents as of 2014. The target of the program is to have at least one graduate from NFELTP per 200,000 population operating a multi-disease surveillance system[24]. In addition to the degree awarding 2-year masters course, NFELTP also offers a series of short courses meant to strengthen the epidemiological capacity of various public health practitioners at the Federal, State and Local Government Areas (LGA) levels. The Program has a broad base of implementing partners, who include the FMOH, FMARD, ABU, UI, CDC and African Field Epidemiology Network (AFENET). All these organizations are represented in a multi-agency Steering Committee that is headed by the FMOH-based Program Director. The Steering Committee meets bi-annually to guide the implementation of the program, evaluate its progress and mobilize resources for the program.

### 1. Vision, Mission and Goal and Multi-year Objectives of the program [24]

**Vision:** NFELTP seeks to become a world class FELTP producing public health leaders and practitioners that can strengthen and lead public health systems to prevent and reduce morbidity and mortality from priority diseases in Nigeria.

**Mission:** NFELTP exists to develop, implement, and strengthen an effective public health surveillance and response system with adequate numbers of competently trained personnel providing epidemiologic service, public health research, and public health emergency response to all Nigerians.

**Goal statement:** Develop and implement a sustainable, effective, networked and adequately staffed multi-disease public health surveillance and response system that is operational in all States and LGAs in Nigeria by 2020. Multi-year objectives; Implement a tiered public health workforce development plan Strengthen IDSR at all levels (Federal, State, LGA, community); Conduct and disseminate public health operations research on priority topics; Develop and implement a sustainability plan and support NFELTP operations

### 2. Course content and field work

Like other FETP/FELTPs, the Nigeria 2-year program consists of formal instruction and service activities. The program supports competencies in four key scientific domains: epidemiology, public health surveillance, biostatistics, and scientific communication. Other minor domains include veterinary epidemiology, preventive effectiveness, leadership and management, teaching and mentoring as well as laboratory skills. These courses are covered in four clusters of training each lasting about 4 to 6 weeks. There are cluster exams at the end of each cluster of training. Before award of the degree, the resident has to complete and defend a protocol-based dissertation. The didactic sessions are augmented by quarterly academic seminars. The program runs three tracks - medical epidemiology, veterinary epidemiology and laboratory epidemiology and management. The distinction between the 3 tracks is reflected in their course content, field posting assignments, degrees awarded as well as potential deployment post-training.

## Progress of the program (2008 – 2014)

Workforce development: This has been achieved through the tiered approach of long course (2 years degree course) and short courses that range from 3-6 months. For the 2 year long course the program has recruited a cohort of residents annually since its inception in 2008. The first three cohorts have successfully completed the 2-year course. For equitable distribution of skilled public health workers the program has ensured recruitment from all regions and states of the country. Of the 207 recruited residents 120 (58%) are medical doctors, 54 (26%) are laboratory scientists and 33(16%) are veterinarians. The target of the program is to train 5 epidemiologists per million populations in Nigeria. In its six years of implementation the program has achieved 27% of its target (range of 36% in North Central to 18% in South South region) (Table 1).

**Table 1:** NFELTP residents by Zone 2008-2014 (N=207)

Zone	Number	Per 1 million population	% Coverage*
North West	42	1.14	**23%**
North Central	44	1.83	**36%**
South West	40	1.21	**24%**
North East	31	1.41	**28%**
South East	32	1.78	**36%**
South South	18	0.9	**18%**
**Total**	**207**	**1.34**	**27%**

Target is – 5 epidemiologist per 1 million population

**Figure 1:**
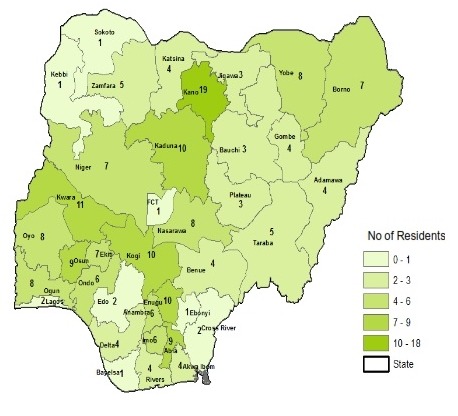
Residents recruitment by State of Origin 2008 – 2014 (N=207)

Each of the state has at least one resident recruited in the program with an average recruitment per state of 5.6 (range of 1-19) (Figure 1).

**Figure 2:**
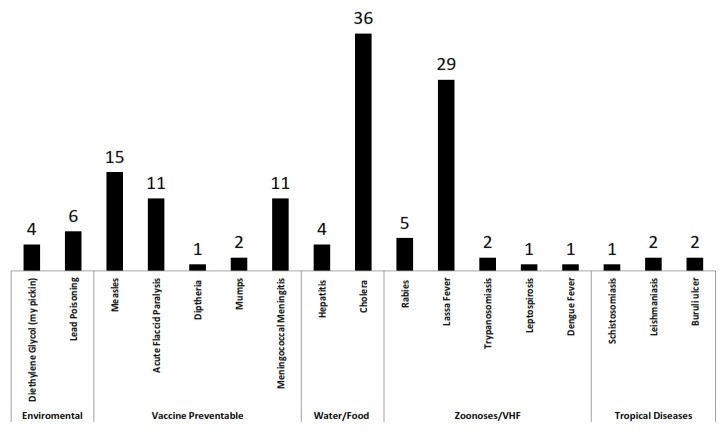
Outbreaks investigated and responded to by residents in NFELTP 2008 - 2014

In 2008, the program recruited 13 residents (6 physicians, 4 veterinarians and 3 laboratorians). In 2011, the program in response to demand for additional residents and graduates, scaled up and recruited 39 residents. The annual recruitment has remained between 40 and 53 with variations in tracks as shown in Figure 2. A total of 595 health care workers have been trained through the short courses. Typically these courses are 3 to 6 months of duration with 2-4 week didactic session and the rest of the time is used to implement a field-based activity. The courses range from HIV program management, monitoring and evaluation, outbreak response and surveillance, vaccine preventable diseases (polio), zoonoses, leadership and management as well as HIV TB collaborations (Table 2).

**Table 2:** Trainees of short courses 2007 – 2014(N=595)

Type of short course	Month/Year	Persons trained	Venue	Comments
HIV/AIDs Program Management 6 month course	September 2013	47	Abuja	Trained on HIV/AIDs epidemiology, surveillance, program management, monitoring and evaluation. Conducted 2 competency-based field projects
HIV competency-based Monitoring and evaluation	July-October 2012	75	Abuja	Competency based monitoring and evaluation of programs with emphasis on HIV. Course done through EFMC
Stop Transmission of Polio Training	June 2012	80		Create vaccine preventable disease capacity particularly polio
Outbreak investigation	November 2011	40	Ibadan	State RRT trained on outbreak investigation – funded by FMOH
Outbreak investigation	September 2011	35	Minna	State RRT trained on outbreak investigation – funded by FMOH
Outbreak investigation	June 2011	33	Kaduna	State RRT trained on outbreak investigation – funded by FMOH
Basic epidemiology course and health leadership and management	January 2011	87	Sokoto	Equipping frontline health care workers with basic epidemiology and health leadership and management skills in northern Nigeria – funded by US Department of Health & Human Services– Health Diplomacy
Zoonoses outbreak and surveillance Short course	September 2010	32	Vom	Zoonoses surveillance and outbreak – USAID funded
Outbreak and surveillance	July –October 2008	35	Minna	State epidemiologists training on outbreak investigation and surveillance with emphasis on vaccine preventable diseases
HIV/TB collaboration (2 courses)	2007/08	66	Zaria, Sokoto	HIV/TB epidemiology and collaboration, data analysis
Outbreak and surveillance (2 courses)	2007/8	70	Enugu Lagos	Emphasis on surveillance and outbreak investigation on influenza and vaccine preventable diseases-supported by GID and USAID
**Total**		**595**		

Funding for these courses has been from multiple sources.

**Response to public health emergencies:** The residents have been involved in response and management infectious and outbreaks including lead poisoning outbreak in Zamfara state, cholera outbreak, cerebrospinal meningitis, Lassa fever and acute renal failure in children as a result of consumption of a contaminated acetaminophen-based teething mixture. Other outbreaks investigated include measles, rabies and Leptospirosis. Overall 133 suspected outbreaks have been responded to ranging from environmental related outbreaks, vaccine preventable diseases, water and food borne, zoonoses, (including suspected viral hemorrhagic fevers) as well as neglected tropical diseases. Some of these outbreaks responded to are described in published articles[14–16, 18, 25] (Figure 2).

One health, emerging infectious diseases: With its emphasis on one health approach of solving public health issues the program has recruited physicians, veterinarians and laboratorians to work jointly on human, animal and environmental health issues. Residents have worked to identify risk factors of disease at the human animal interface for influenza, brucellosis, tick-borne relapsing fever, rabies, Leptospirosis, zoonotic helminthic infections. In addition to zoonoses short courses for multi-displinary teams the program has been involved in improving data analysis, surveillance systems and risk factors for leading zoonoses in Nigeria. The program worked with CDC recently to identify potential for emerging infectious diseases in a traditional ceremony that involves bats in southwest Nigeria [18, 19, 25–28].

**Program evaluation an d surveillance:** The residents have supported scale up of IDSR capacity at the federal and state levels through training, data analysis and dissemination. The program is building the public health workforce to operate multi-disease surveillance systems at all levels of administration. Residents help analyze surveillance data, evaluate surveillance systems and disease control programs. To provide decision makers with evidence for program implementation a number of residents have looked at hospital based mortality to identify leading causes of death. Evaluation of IDSR at peripheral levels has identified specific gaps that need to be addressed to ensure robust surveillance system and timely effective response to public health emergencies [10, 21, 29, 30].

**Disease control efforts:** The program is also working on other disease specific control efforts including HIV to support program evaluation for improvement. Residents are currently working on data analysis, assessment of HIV risk factors for most at risk populations (e.g. prisoners, Men who have Sex with Men, Commercial Sex Workers and Commercial drivers). Each year at least three residents work on malaria related projects involving supporting surveillance data analysis, surveillance evaluation, and rapid test kits validation [31].

**Involvement in polio eradication initiative:** The program has created a National Stop Transmission of Polio (NSTOP) to support the Nigeria Federal Government's efforts in the polio eradication initiative (PEI) as part of the surge capacity of the National Polio Eradication Emergency Plan. NSTOP initially was created to strengthen government efforts in reaching underserved population with basic primary health care needs such as vaccination. In addition the NSTOP support supplemental immunization activities, surveillance, routine immunization and research on priority questions to guide program implementation. NSTOP is run by graduates and residents of the program and this has allowed them to build local capacity and leadership for PEI at the state and local government areas in thematic areas such as micro planning, supplemental immunization activity, data analysis, vaccine cold chain management, demand creation, informatics (use of smart phones and Geographic Information System to report disease and monitor implementations) and surveillance. The program has supported activities of the National and State Polio Emergency Operation Centers. As a result of the NSTOP over 1 million children < 5 years have been reached in underserved populations[32, 33].

**Setting priority research agenda:** In an effort to support evidence for public health decision making, the program has worked with the FMOH to identify research priorities. Operational research workshops have been held to identify research priorities for HIV, malaria and polio. Some residents are working on non-communicable disease including hypertension, obesity and cancer. A resident assessed cervical cancer knowledge, screening and service utilization as well as predicators of precancerous changes in one of the states [34]. Residents are also working on identifying prevalence and risk factors of other non-communicable diseases such as hypertension and obesity. A research prioritization workshop will be held to guide implementation in NCD research in the country.

**Conferences and presentations:** The residents have participated in national, regional, and international conferences and won numerous accolades for their works. The conferences include the annual EIS conference, the global Training in Public Health Intervention Network (TEPHINET), regional AFENET and others. Over 200 papers have been presented at these conferences. A second cohort resident became the first resident from an African FELTP to win the prestigious William Foege Award for best abstract and presentation during the international night of 2012 EIS conference. His work on tuberculosis has subsequently been published [35]. NFELTP has won best presentations in numerous conferences both locally and internationally.

**Networking:** During the 2012 flooding an emergency operation center was created by the Federal Ministry of Health to coordinate surveillance and response to the flooding in the country. The flooding affected 15 states. The residents were involved in post flood assessment, response to health emergencies and dissemination of information. The program is a member of the AFENET, TEPHINET and African Program for Advanced Research Epidemiology Training (APARET) which allows its residents and graduates to network with other programs. With creation of the Nigeria Centre for Disease Control (NCDC), the residents and graduates will serve as the frontline public health workers to operate surveillance and response in the ssame way other similar programs have provided these public health services to their national public health institutes. NCDC was created in 2010 to coordinate surveillance and response functions of the FMOH. Creation of such public health institutes as the NCDC is a global best practice and one of the priorities in the global health security agenda. Graduates have successfully applied and won grants from a number of organizations including the Bill Melinda Gates Foundation [36].

## Discussion

The commencement of NFELTP was a novel approach to building sustainable epidemiological capacity to strengthen public health systems especially surveillance and response systems in Nigeria. The program has offered public health service in public health emergency response and strengthening surveillance systems. Joint training in class and field activities is bearing fruits in strengthening collaborations between the human and animal health sectors and may be an important platform for addressing zoonoses in the country. TraininThe commencement of NFELTP was a novel approach to building sustainable epidemiological capacity to strengthen public health systems especially surveillance and response systems in Nigeria. The program has offered public health service in public health emergency response and strengthening surveillance systems. Joint training in class and field activities is bearing fruits in strengthening collaborations between the human and animal health sectors and may be an important platform for addressing zoonoses control and prevention in the country. Training and capacity building efforts should be tied to specific system strengthening and not viewed as an end to them. The approach of linking training and service provision may be an innovative approach towards addressing the numerous health challenges. Within 6 years Nigeria has come from very few field epidemiologists to 65 graduates and 142 current trainees from all 36 states, comprising 120 medical epidemiologists, 33 veterinary epidemiologists, 54 public health laboratory scientists trained jointly to address public health problems in Nigeria in the 21st century using new evidence-based methods. This approach has begun bearing fruit, for example the early detection and prompt effective response in teething mixture contamination and lead poisoning outbreak likely saved lives, and the current achievements of Nigeria in polio eradication cannot be separated from the existence of NFELTP's National Stop Transmission of Polio. Indeed certification of Nigeria as Guinea worm free was achieved under the auspices of a program director who is a proud graduate of NFELTP's 2nd cohort in 2013.

However there are systemic issues such as sustainable funding, career progression and retention that may urgently need to be addressed to reap the full benefits of this initiative. There are additional challenges beyond human resource development that need to be addressed such as health financing, governance and leadership and commodities and supplies that holistically need to be strengthened. NFELTP is serving an unmet need for public health workforce to operate surveillance and response for infectious diseases and non-infectious. Involving NFELTP has improved response to outbreak particularly thorough investigation, laboratory support and rapid effective response hence reducing morbidity and mortality from leading causes of death. Even though the program is funded by disease specific resources it is contributing to workforce development for the specific disease control efforts - HIV, malaria, polio, but also health systems strengthening. All these need skilled workforce for effective implementation. The One Health approach has helped in bridging the communication and collaboration gaps in addressing surveillance and response required for zoonoses, emerging infectious diseases and food safety. It's also an important approach in the global health security agenda, IDSR and IHR.

Operational research by residents has guided implementation of programs. Residents have demonstrated increased efficiencies by use of new technologies in disease control such as use of smart phones, Open Data Kit and are involved in assessments for implementations of District Health Information Systems (DHIS2) a platform that has been adopted to improve health information systems in Nigeria and other African countries. NFELTP is a Multi-agency collaboration that is addressing the critical need for skilled public health worker force for health system strengthening and ensuring institutional system strengthening and a culture of evidence based decisions. Some challenges including sustainable funding, coordination and insecurity, appropriate deployment have hampered realization of the full potential of the program. A strategic plan has been developed to guide implementation.

## References

[1] World Health Organization (2007). Everybody's business: strengthening health systems to improve health outcomes. WHO's framework for Action.

[2] Saraladevi Naicker, Jacob Plange-Rhule, Roger Tutt C (2009). Shortage of healthcare workers in developing countries. Ethn Dis.

[3] Kariuki Njenga M, Traicoff D, Tetteh C, Likimani S, Oundo J (2008). Laboratory Epidemiologist: Skilled Partner in Field Epidemiology and Disease Surveillance in Kenya. J Public Health Policy.

[4] Nsubuga P, Nwanyanwu O, Nkengasong J, Mukanga D, Trostle M (2010). Strengthening public health surveillance and response using the health systems strengthening agenda in developing countries. BMC public health..

[5] Nsubuga P (2011). Field Epidemiology and Laboratory Training Programs in sub-Saharan Africa from 2004 to 2010: need, the process, and prospects. Pan African Medical Journal..

[6] Rolle IV, Pearson ML, Nsubuga P (2011). Fifty-five years of international epidemic-assistance investigations conducted by CDC's disease detectives. American journal of epidemiology..

[7] Becker KM (2012). Field Epidemiology and Laboratory Training Programs in West Africa as a model for sustainable partnerships in animal and human health. J Am Vet Med Assoc..

[8] Nguku P (2010). An investigation of a major outbreak of Rift Valley fever in Kenya: 2006-2007. American journal of tropical medicine and hygiene..

[9] Dooyema C (2012). Outbreak of Fatal Childhood Lead Poisoning Related to Artisanal Gold. Environmental Health Perspectives..

[10] Abubakar A (2013). Assessment of integrated disease surveillance and response strategy implementation in selected Local Government Areas of Kaduna state. Annals of Nigerian Medicine..

[11] Koo D, Thacker S (2010). In snow's footsteps: Commentary on shoe-leather and applied epidemiology. American journal of epidemiology..

[12] López A, Cáceres VM (2008). Central America Field Epidemiology Training Program (CA FETP): a pathway to sustainable public health capacity development. Hum Resour Health..

[13] Ortiz JR, Katz MA, Mahmoud MN, Ahmed S, Bawa SI (2007). Lack of Evidence of Avian-to-Human Transmission of Avian Influenza A (H5N1) Virus among Poultry Workers, Kano, Nigeria, 2006. J Infect Dis..

[14] Akyala Ishaku A, Bright Esyine Shadrack, Olufemi Ajumobi (2014). Investigation of Cholera Outbreak in an Urban North Central Nigerian Community-The Akwanga Experience. Public Health Research.

[15] Kabiru Ibrahim Getso, Idris Suleman Hadejia, Kabir Sabitu, Patrick Mboya Nguku (2013). Prevalence and Determinants of Childhood Lead Poisoning in Zamfara State, Nigeria. Journal of health and pollution.

[16] Akyala Ishaku, Olufemi Ajumobi, Adebola Olayinka (2013). Implication of coliforms as a major public health problem in Nigeria. Journal of Public Health and Epidemiology.

[17] Lo YC, Dooyema CA, Neri A, Durant J, Jefferies T (2012). Childhood Lead Poisoning Associated with Gold Ore Processing. Environ Health Perspect..

[18] Awosanya JP, Nguku A, Oyemakinde Omobowale O (2013). Factors associated with probable cluster of leptospirosis among kennel workers in Abuja, Nigeria. Pan African medical journal..

[19] Aworh MK, Okolocha E, Kwaga J, Fasina F (2013). Human brucellosis: seroprevalence and associated exposure factors among abattoir workers in Abuja, Nigeria – 2011. Pan African medical journal..

[20] Vora NM, Osinubi M, Wallace RM, Aman-Oloniyo A, Gbadegesin YH (2014). Assessment of Potential Zoonotic Disease Exposure and Illness Related to an Annual Bat Festival - Idanre, Nigeria. MMWR Morb Mortal Wkly Rep..

[21] Abubakar A, Idris S, Sabitu K, Shehu A, Sambo M (2010). Emergency preparedness and the capability to identify outbreaks: A case study of Sabon Gari Local Government Area, Kaduna state. Annals of Nigerian Medicine..

[22] Kasolo F, Yoti Z, Bakyaita N, Gaturuku P, Katz R, Fischer JE, Perry HN (2013). IDSR as a platform for implementing IHR in African countries. Biosecurity and bioterrorism..

[23] Sow I, Alemu W, Nanyunja M, Duale S, Perry HN, Gaturuku P (2010). Trained district health personnel and the performance of integrated disease surveillance in the WHO African region. East Afri J Public Health..

[24] Nigeria Field Epidemiology and Laboratory Training Programme Nigeria Field Epidemiology and Laboratory Training Program Strategic Plan, 2014 to 2020. http://www.nigeria-feltp.net.

[25] Adeoye OA, Aman-oloniyo P, Nguku A, Oduneye, Dawodu M (2013). Case Based Surveillance for Measles in Lagos, South Western Nigeria.

[26] Ekong PR, Juryit, Dika NM, Nguku P, Musenero M (2012). Prevalence and risk factors for zoonotic helminth infection among humans and animals - Jos, Nigeria, 2005-2009. Pan African medical journal.

[27] Kamani J, Baneth G, Mumcuoglu KY, Waziri NE, Eyal O, Guthmann Y, Harrus S (2013). Molecular detection and characterization of tick-borne pathogens in dogs and ticks from Nigeria. PLoS neglected tropical diseases..

[28] Aworh MK, Nwosuh CI, Ajumobi OO, Okewole PA (2010). A Retrospective Study of Rabies Cases Reported at Vom Christian Hospital, Plateau State, Nigeria, 2006 - 2010. Nigerian Veterinary Journal.

[29] Preacely N, Biya O, Gidado S, Ayanleke H, Kida M (2012). Hospital-Based Mortality in Federal Capital Territory Hospitals-Nigeria, 2005 - 2008 Short communication. Pan African medical journal.

[30] Bashorun A, Ahumibe A, Olugbon S, Nguku P, Sabitu K (2013). Evaluation of Cholera and Other Diarrheal Disease Surveillance System, Niger State, Nigeria-2012.

[31] Elizabeth Adedire, Asekun-Olarinmoye Esther O (2013). Caregivers Home-Care Practices Towards Childhood Febrile Illnesses In Urban And Rural Areas Of Osun State, Nigeria. International Journal of innovative research and Studies.

[32] Ewen Callaway (2013). Polio's moving target. Nature.

[33] Centers for Disease Control and Prevention (CDC) (2013). Polio Field Census and Vaccination of Underserved Populations - Northern Nigeria, 2012-2013. MMWR Morb Mortal Wkly Rep.

[34] Adeoye O, Fawole O, Ajayi I, Nguku P (2013). Cervical Cancer Knowledge, Screening Service Utilization and Predictors of Precancerous Cervical Changes: A Population Based Survey of Sexually Active Women in Lagos, South Western Nigeria.

[35] Ibrahim LM, Hadejia IS, Nguku P, Dankoli R, Waziri NE (2014). Factors associated with interruption of treatment among Pulmonary Tuberculosis patients in Plateau State, Nigeria. Pan African Medical Journal..

[36] Grand Challanges in Global Health http://www.grandchallenges.org/explorations/Pages/grantsawarded.aspx?Round=all&Phase=all&ProjectID=1243.

